# Characterization and Biological Activity of a Novel Exopolysaccharide Produced by *Pediococcus pentosaceus* SSC–12 from Silage

**DOI:** 10.3390/microorganisms10010018

**Published:** 2021-12-23

**Authors:** Yang Fan, Xinqin Li, Rong Tian, Ruxue Tang, Jianguo Zhang

**Affiliations:** 1 South Pratacultural Center, South China Agricultural University, Guangzhou 510642, China; 18062993638@163.com (Y.F.); lxinqin1022@163.com (X.L.); tianrong0929@163.com (R.T.); byndtrx@163.com (R.T.); 2 Guangdong Engineering Research Center for Grassland Science, Guangzhou 510642, China

**Keywords:** antioxidant, antibacterial, exopolysaccharides, *Pediococcus pentosaceus*, silage

## Abstract

In this study, 22 strains of exopolysaccharides-producing lactic acid bacteria were isolated from silage, and the strain SSC–12 with the highest exopolysaccharide (EPS) production was used as the test strain. The SSC–12 was identified as *Pediococcus pentosaceus*, based upon 16S rDNA gene sequencing and Neighbor Joining (NJ) phylogenetic analysis. The analysis of the kinetic results of EPS generation of SSC–12 showed that the EPS generation reached the maximum value at 20 h of culture. The characterization study showed the EPS produced by SSC–12 was a homogeneous heteropolysaccharide comprising glucose (42.6%), mannose (28.9%), galactose (16.2%), arabinose (9.4%), and rhamnose (2.9%). The EPS had good antioxidant activity, especially the activity of scavenging hydroxyl free radicals. At the same time, the EPS also had strong antibacterial ability and could completely inhibit the growth of *Staphylococcus aureus*. The EPS produced by the *Pediococcus pentosaceus* SSC–12 can be used as a biologically active product with potential application prospects in the feed, food, and pharmaceutical industries.

## 1. Introduction

Exopolysaccharide (EPS) is a carbohydrate secreted by microorganisms during growth and metabolism. It is produced by several types of microorganisms that produce EPS, such as species of *Lactobacillus* [1], *Bacillus* [2], *Bifidobacterium* [3], *Leuconostoc* [4], and *Pediococcus* [5]. Among them, lactic acid bacteria (LAB) are generally recognized as safe (GRAS) and food-grade microorganisms. Previous studies have shown that most *Lactobacillus* spp., such as *L. Kefiranofacience* [6], *L. plantarum* [1], *L. confuses* [5], and *L. acidophilus* [7], can produce EPS. 

EPS can be divided into homopolysaccharides (HoPSs) and heteropolysaccharides (HePSs) based on its composition [8]. HoPSs are composed of repeating units of monosaccharide—primarily glucose, galactose, or rhamnose [9]; contrarily, HePSs are polysaccharides comprising three or more monosaccharides, such as arabinose, rhamnose, galactose, and mannose [10].

The EPS produced by different LAB have different bioactive functions. It has been identified that EPS produced by LAB has immunomodulatory properties, antioxidant activity, antibacterial activity, anti-tumor, anti-cancer, hypoglycemic, and other biological properties [11]. EPS produced by LAB could enhance cellular defense mechanisms and prevent diseases by reducing reactive oxygen species and free radicals [12]. Therefore, it is a safe and harmless biologically active substance that has potential antioxidant property. Moreover, EPS produced by LAB could inhibit the growth of harmful microorganisms and even kill them, thereby preventing the occurrence of microbial infections [6]. Therefore, EPS can be used as an alternative to antibiotics to reduce drug resistance. With the increasing health hazards caused by food spoilage or bacterial infections, the search for EPS with antioxidant and antibacterial activities has attracted the attention of researchers.

Currently, the sources of EPS-producing LAB are relatively limited; hence, the identification of new sources and the determination of their functional activities have become the focus of several studies. Silage is a kind of roughage obtained through the fermentation of LAB under anaerobic conditions to inhibit the reproduction of various miscellaneous bacteria [13]. Silage is rich in a variety of LAB, and hence it is potentially a good source of LAB. This study primarily aimed to screen out LAB with high EPS yield from silage, analyze the relationship between LAB growth and EPS production, and determine monosaccharide composition and biological activity of EPS produced. In this study, we not only identify a new source of EPS-producing LAB, but also provide theoretical support for EPS application in the feed, food, and pharmaceutical industries.

## 2. Materials and Methods

### 2.1. Screening and Identification of EPS-Producing LAB

LAB was isolated from silage and purified by culturing on De Man, Rugose, and Sharpe (MRS) agar medium under anaerobic condition for 24–48 h at 37 °C [1]. After morphological observation, a single colony, having milky white color, sticky surface, surrounding diffusion phenomenon, protruding round shape, and obvious viscosity when picked by inoculation ring [11], was suspected to be the EPS-producing LAB. 

The above strains were inoculated into 50 mL MRS liquid medium and statically cultured at 37 °C for 24 h. Next, the broth was collected, centrifuged at 8000 r/min for 10 min, and the supernatant was collected, followed by the addition of 95% ethanol (3 × volume) and incubation at 4 °C overnight [1]. The mixture was centrifuged at 8000 r/min for 10 min, and the precipitate was collected and dissolved in distilled water, followed by dialysis for 3 days to obtain crude EPS solution [14]. The EPS production was measured by phenol sulfuric acid method, and the strains with the highest EPS production were identified.

The screened strains were purified and cultured for two generations to obtain bacterial suspension, and total DNA was extracted from cell precipitate using TIANamp Bacteria DNA Kit. The 16S rDNA was amplified with primer pair 27F/1492R using polymerized chain reaction (PCR) procedure [15]. Purified PCR products purified from each strain were sequenced by ABI3730–XL DNA Analyzer, and we used the Blast (http://www.ncbi.nlm.nih.gov.blast, (accessed on 13 May 2021)) to compare the spliced sequence file with the data in the NCBI 16S database [6]. The MEGA–X software was used to construct phylogenetic evolutionary tree, and the species with more than 99% similarity were identified. The identified LAB was stored in MRS liquid medium with 20% glycerol at −80 °C [16].

### 2.2. Analysis of Strain Growth and EPS Production

The identified strains were inoculated into MRS liquid medium and cultured for two generations. After the LAB was cultured for different periods, the number of LAB was counted by the plate counting method and EPS production was determined by using the phenol sulfuric acid method. The peak time for EPS production was determined, and the extraction and purification of EPS was carried out. The purification steps of EPS were repeated to obtain EPS aqueous solution. The EPS solution was freeze-dried (Thermo Modulyo Freeze Dryer, Thermo Fisher Scientific, Waltham, MA, USA) at low temperature, and the flocs were stored at ambient temperature in a sealed state [16].

### 2.3. Molecular Weight (Mw) Determination of EPS

The uniformity of EPS was determined with a Gel Permeation Chromatography (GPC, Wyatt, Santa Barbara, CA, USA) equipped with an Agilent PL aquagel–OH MIXED–H column (10μm, 300 × 7.5 mm) and an evaporative light-scattering detector. EPS solution (2 mg/mL), 100 μL, was injected and eluted with redistilled water at a flow rate of 1 mL/min [15]. According to the peak time of the sample, the software calculated the molecular weight of the sample.

### 2.4. Determination of EPS Monosaccharide Composition 

After the EPS samples were acid hydrolyzed, their monosaccharide composition was analyzed by high performance anion exchange chromatography (HPAEC) (DIONEX ICS-6000, Thermo Fisher Scientific, Waltham, MA, USA) equipped with Dionex™ CarbopacTM PA-20 anion exchange chromatography column (3 × 150 nm) and electrochemical detector. NaOH (5 mmol) was used as the mobile phase with a flow rate of 0.4 mL/min at 30 °C [17]. Established calibration curves of mannose, rhamnose, glucose, galactose, and arabinose were used for quantitative analysis [18].

### 2.5. Fourier Transform-Infrared (FT-IR) Spectroscopic Analysis of EPS

The characteristic functional group in EPS were determined by FT-IR spectroscopy. One milligrams of dry EPS were taken and compressed with 100 mg of dry KBr, scanned in the range of 4000 cm^−1^ to 400 cm^−1^ and recorded the infrared spectrum.

### 2.6. Assessment of EPS Antioxidant Activity

#### 2.6.1. DPPH Radical Scavenging Ability

The 2,2-diphenyl-1-picrylhydrazyl (DPPH) radical scavenging ability of EPS was measured according to the method described by Rajoka et al. [19], with minor modifications. The reaction solution consisted of 2.0 mL of water, 1.0 mL of EPS sample (1.0–10.0 mg/mL), and 0.4 mL of DPPH ethanol solution (0.5 mM). The mixture was agitated and incubated in the dark for 30 min, followed by the measurement of the absorbance at 517 nm. Different concentrations of ascorbic acid in same volume (1.0–10.0 mg/mL) were used as a positive control. Each treatment was carried out three times. The DPPH radical scavenging ability of EPS was calculated as follows (1):

DPPH radical scavenging ability (%) = [1 − (A_11_ − A_01_)] × 100 (1)

where “A_11_” is the absorbance of the sample, “A_01_” is the absorbance of the control group, and “A_10_” is the absorbance of the blank. Ethanol (0.4 mL) and sample solutions of different dilutions (1.0 mL) were used as the control, and DPPH radical-ethanol (0.4 mL) and water (1.0 mL) were used as the blank.

#### 2.6.2. Hydroxyl Radical Scavenging Ability

The hydroxyl radical scavenging ability of EPS was measured according to the method described by Xu et al. [15] with few modifications. Briefly, 50 μL PBS solution (20 Mm, pH 7.4), 25 μL 1,10–phenanthroline solution (12.5 mM), 25 μL FeSO_4_ solution (2.5 mM), and 25 μL H_2_O_2_ solution (20 mM) were added consecutively in a test tube. Next, 100 μL EPS of different concentrations (1.0–10.0 mg/mL) was added to the mixture, followed by incubation at 37 °C for 1 h and measurement of absorbance at 536 nm. Different concentrations of ascorbic acid in equal volume (1.0–10.0 mg/mL) were used as positive control. The experiment was carried out in triplicate. The hydroxyl radical scavenging ability of EPS was calculated as follows Equation (2):

Hydroxyl radical scavenging ability (%) = (A_sample_ − A_black_)/(A − A_black_) × 100 (2)

where “A_sample_” was the absorbance when the sample contains different concentrations of EPS, “A_black_” was the absorbance of the sample when EPS and H_2_O_2_ were replaced by water, and “A” was the absorbance of the sample when EPS and H_2_O_2_ were replaced by water.

#### 2.6.3. Superoxide Radical Scavenging Activity 

The superoxide radical scavenging activity of EPS was measured according to the method described by Xu et al. [15]. The superoxide radical was generated in 50 μL of Tris-HCl buffer (pH 8.0, 150 mM) containing 25 μL of pyrogallol (1.50 mM, dissolved in 10 mM HCl) and 100 μL of EPS samples (1.0–10.0 mg/mL). Next, the mixture was incubated at 25 °C for 30 min and the absorbance was measured at 325 nm. Different concentrations of ascorbic acid in equal volumes (1.0–10.0 mg/mL) were used as a positive control. Each experiment was carried out in triplicate. The superoxide radical scavenging activity of EPS was calculated as follows Equation (3):

Superoxide radical scavenging activity (%) = [1 − (A_s_ − A_c_)/(A_b_ − A_0_)] × 100 (3)

where “A_s_” is the absorbance of samples containing EPS and pyrogallic acid, “A_c_” is the absorbance of samples containing EPS but were used 10 mM HCl was used instead of pyrogallic acid, “A_b_” is the absorbance of deionized water instead of EPS but containing pyrogallic acid, “A_0_” is the absorbance of EPS sample and pyrogallic acid sample replaced with deionized water and 10 mM HCl, respectively.

#### 2.6.4. Reducing Ability

The reduction ability of EPS was measured according to the method of Rajoka et al. [19]. A solution containing 1.5 mL sodium phosphate buffer (0.2 M, pH 7.3), 1.5 mL K_3_Fe(CN)_6_ (1%, *w*/*v*), then 1.5 mL EPS samples (1.0–10.0 mg/mL) was incubated at 50 °C for 25 min. After cooling, 1.5 mL trichloroacetic acid (12%, *w*/*v*) was added, and the mixture was centrifuged (6000× *g*/min, 4 °C, 10 min). Next, 0.5 mL FeCl_3_ (0.2%, *w*/*v*) was added to the supernatant, and the absorbance was measured at 700 nm. Different concentrations of ascorbic acid in equal volume (1.0–10.0 mg/mL) were used as a positive control. The experiment was carried out in replicates. The reducing ability of EPS was calculated as follows Equation (4):

Reducing ability = A_1_ − A_0_ (4)

where “A_1_” is the absorbance of EPS sample, “A_0_” is the absorbance of FeCl3 replaced by deionized water.

### 2.7. Measurements of EPS Antibacterial Ability

In this experiment, *Staphylococcus aureus* GDMCC 1.1220, *Salmonella enterica* subsp. *enterica* GDMCC 1.345, *Listeria monocytogenes* GDMCC 1.347 were selected as indicator bacteria. These bacteria were separately inoculated into LB broth medium and cultured at 37 °C for 24 h with shaking, which was repeated twice to obtain the second-generation strain [1]. Next, the bacterial suspension was adjusted to an estimated concentration of 10^5^−10^6^ colony forming units (CFU)/mL based on the absorbance at 600 nm. The purified EPS was diluted with deionized water into EPS solutions of different concentrations (1.0–10.0 mg/mL), followed by filtration and sterilization with a 0.45 μM microporous filter. Next, 1 mL of the EPS solutions of different concentrations and 10 μL of indicator bacteria solution were added to 1 mL of fresh LB broth medium and cultured for 24 h at 37 °C with shaking [6], with equal amount of sterile water used as a blank instead of EPS. The inhibition rate and bacterial count indicated the antibacterial activity of EPS. The experiment was carried out in triplicate. The formula for the inhibition rate is as follows Equation (5):

Inhibition rate (%) = (1 − log ^AEPS^/log ^ABlack^) × 100 (5)

where “AEPS” is the number of colonies of EPS sample, “ABlack” is the number of colonies of positive control.

### 2.8. Statistical Analysis

All statistical analyses were conducted using IBM SPSS Statistics 22 software for Windows (IBM Corp, New York, NY, USA), and means were compared for significance by Duncan’s multiple range method.

## 3. Results and Discussion

### 3.1. EPS-Producing LAB and EPS Yield

**Figure 1:**
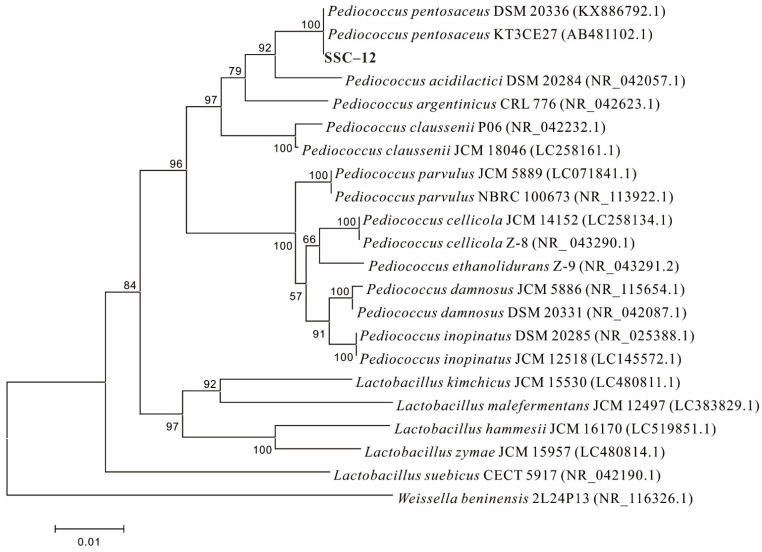
Neighbor-joining phylogeny of SSC–12 with closely related *Lactobacillus* strains according to 16S rRNA gene sequence.

In this experiment, 22 EPS-producing LAB strains were screened out after the observation of colony morphology, Gram stain test, and EPS yield determination. As shown in Table 1, the EPS yield of 22 strains screened from silage ranged 30.5–276.6 mg/L. Of all the strains, 15 were considered as high-yield EPS strains (EPS yield > 100 mg/L), based on Smitinont [20]. SSC–12 exhibited the highest yield of crude EPS (SSC–12 EPS), producing 276.6 mg/L in De Man, Rugose, and Sharpe (MRS) broth; therefore, this strain was selected for further studies. The EPS yield of SSC–12 selected in this experiment was higher than that of *Pediococcus pentosaceus* F3 (99.53 mg/L) [21], but was similar to that produced by *P. pentosaceus* NR 042058.1 (263.6 mg/L) [22]. The 16S rDNA results indicated that SSC–12 was closely related to *P. pentosaceus* (>99% identity); it clustered apart from other species of this genus and, thus, was identified as *P. pentosaceus* (Figure 1). *Pediococcus pentosaceus* is a homofermentative LAB with physical characteristics and biological functions, which can be used in the production of fermented food [23]. Previously, it has been demonstrated that the EPS produced by *P. pentosaceus* had good antioxidant activity and could be used as food preservative and therapeutic agent.

### 3.2. EPS-Producing LAB and EPS Yield

**Figure 2:**
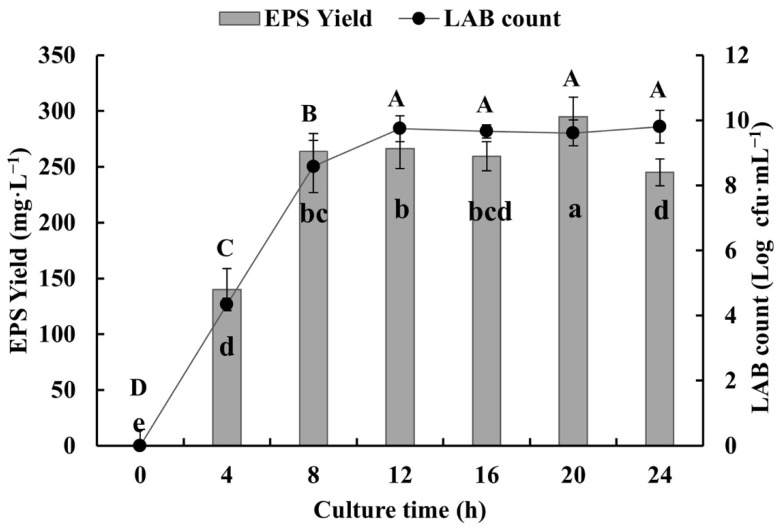
The dynamic relationship between the SSC–12 counts and its EPS yield. The values are expressed as mean ± SD of three independent analyses. Different lowercase letters indicate significant differences among the EPS yields (*p* < 0.05). Different capital letters indicate significant differences among LAB counts (*p* < 0.05).

With increase in culture time, the number and the EPS production of SSC–12 gradually increased and both reached the peak at 20 h of culture (Figure 2). The rapid growth of SSC–12 in the first 8 h of inoculation indicated that it had reached the exponential stage and EPS production increased accordingly. After 12 h of culture, SSC–12 entered a stable stage of slow and stable growth, and its EPSs were relatively stable, and EPS production reached the highest at 20 h. According to previous studies, *L. reuteri* SHA101 and *L. vaginalis* SHA110 reached maximum EPS production in 48 h [24] and *L. plantarum* WLPL04 in 24 h [25]. However, SSC–12 achieved maximum EPS production in 20 h, suggesting that SSC–12 would have a great application value.

### 3.3. Molecular Weight of EPS

The results of gel chromatography showed that the molecular weight of the SSC–12 EPS was 7.6 × 10^4^ Da and lower than *P. pentosaceus* NR 042058.1 [22]. Previous studies have shown that the molecular weights of many HePSs is between 1 × 10^4^ Da and 6 × 10^6^ Da [26]. For example, the molecular mass of *L. coryniformis* NA-3 isolated from Chinese sauerkraut was 8.6 × 10^6^ Da [15]. In this work, the SSC–12 EPS had slightly lower molecular weight, but it was also within the range previously reported. As the basic characteristic of EPS, molecular weight has a certain influence on its physical characteristics and biological activity function [5].

### 3.4. Monosaccharide Composition of EPS

High-performance anion exchange chromatography (HPAEC) determination showed that SSC–12 EPS was a heteropolysaccharide. The total sugar content of SSC–12 EPS was 73.6%, comprising glucose (42.6%), mannose (28.9%), galactose (16.2%), arabinose (9.4%), and rhamnose (2.9%) (Table 2). According to the number of monosaccharide and the proportion of each monosaccharide, EPS produced by SSC–12 was different from that produced by *P. pentosaceus* as previously reported. For example, the EPS produced by *P. pentosaceus* M41 consisted of glucose (79.0%), mannose (9.5%), arabinose (6.2%), and galactose (5.2%) [24], and the EPS produced by *P. pentosaceus* DPS comprised glucose, mannose, and fructose in different ratios [25]. Therefore, SSC–12 produced a novel type of EPS. EPS produced by the same strains might also be composed of different monosaccharides. The monosaccharide composition of EPS is species- and strain-dependent [24], but it is also affected by various factors such as culture medium and culture conditions [11]. 

### 3.5. Fourier Transform Infrared (FT-IR) Analysis of EPS

**Figure 3:**
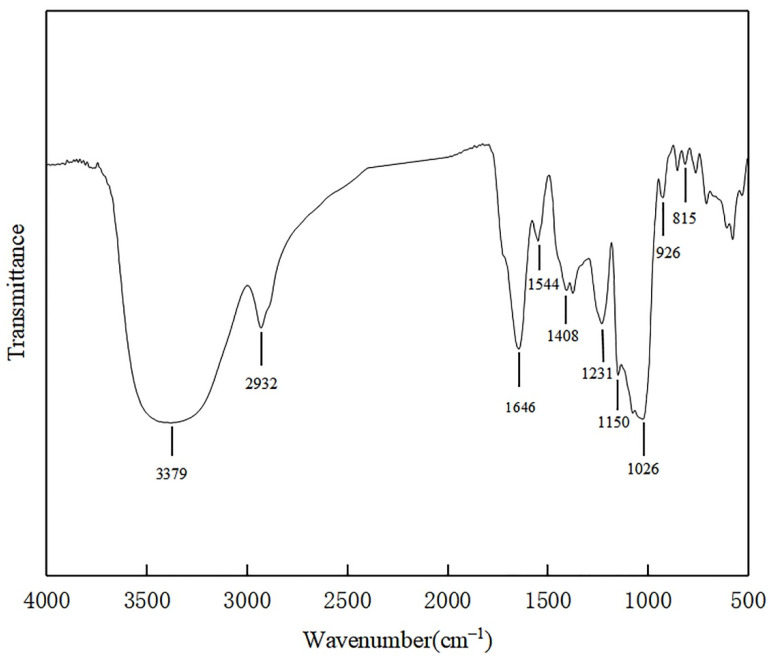
FT-IR spectrum of SSC–12 EPS.

The FT-IR spectrum of SSC–12 EPS showed a complex pattern of peaks from 4000 cm^−1^ to 400 cm^−1^ (Figure 3).The results showed that a wide stretching peak at 3379 cm^−1^ was the stretching vibration absorption peak of hydroxyl group (-OH), and the absorption peak of C-H was located at 2932 cm^−1^, and the absorption peak in this region was the characteristic peak of polysaccharide [27]. There was no obvious peak at 1726 cm^−1^, indicating that there was no uronic acid. The strong band at 1646 cm^−1^ was attributed to the stretching vibration of mannose or galactose [28]. The signal at 1544 cm^−1^ was attributed to the presence of an amide group (N-H) indicating a possible protein binding [29]. The band at 1408 cm^−1^ and 1026 cm^−1^ were ascribed to the bending vibration of the O-H bond and the C-O-C linkage, respectively [30]. The strong frequency band that ranged from 1160 cm^−1^ to 950 cm^−1^ was caused by the stretching vibration of pyran ring and was an ideal fingerprint of EPS [24]. The 926 cm^−1^ might be caused by the asymmetric stretching vibration of the glucose ring, and there might be β-configuration of the sugar units [27]. The peak at 815 cm^−1^ indicated that there was a characteristic absorption peak of mannose [31], which was consistent with monosaccharides composition of SSC–12 EPS.

### 3.6. Antioxidant Activity of EPS

Free radicals, such as reactive oxygen species, combined with biological macromolecules in the body to cause tissue damage and induce different diseases [32]. Certain EPSs can scavenge active oxygen free radicals in vivo and reduce the incidence of diseases. EPS from *L. kimchi* SR8 significantly improved the liver index, serum superoxide dismutase activity, and the survival rate of mice [33]. Moreover, some EPSs have antioxidant properties and can slow aging and deterioration by fighting off excess free radicals in vitro; for example, EPS produced by *P. pentosaceus* has good antioxidant activity and could prolong the shelf life of bananas [21]. The EPS produced by LAB is a natural and safe antioxidant, which could have a good application prospect in food preservation and health product industry. 

DPPH radicals can accept free electrons into stable molecules, thus attacking cells and causing lesions [34]. Hydroxyl free radical is the most active free radical, which could cause oxidative damage to neighboring biological molecules and induce diseases [35]. Superoxide free radicals cause severe tissue damage by inducing lipid peroxidation and oxidative damage [36]. Antioxidants provide electrons to scavenge free radicals through their reducing action, and a high reducing power indicates a strong antioxidant power.

In this study, the antioxidant activity of SSC–12 EPS was assessed in terms of its ability to inhibit the formation of free radicals and its reducing ability. The antioxidant capacity of SSC–12 EPS was concentration-dependent and the gap between them at the same concentration became smaller and smaller. At 10 mg/mL, the DPPHscavenging ability (77.4%), hydroxyl radicalscavenging ability (97.5%), superoxide radicalscavenging ability (77.5%), and reducing ability (1.3) of SSC–12 EPS reached maximum values. Rajoka et al. [19] observed lower values than that of SSC–12 EPS (56.7%) at a concentration of 4 mg/mL, with the superoxide radical scavenging rate of 40.5% and 25.5% for the EPS produced by *L. reuteri* SHA101 and *L. vaginalis* SHA110, respectively. Seo et al. [37] also evaluated the antioxidant capacity of an EPS produced by *L. plantarum* YML009 and reported a lower DPPH radical scavenging (7.2%) and reduction (0.15) activities at 10 mg/mL, compared with those of SSC–12 EPS.

**Figure 4:**
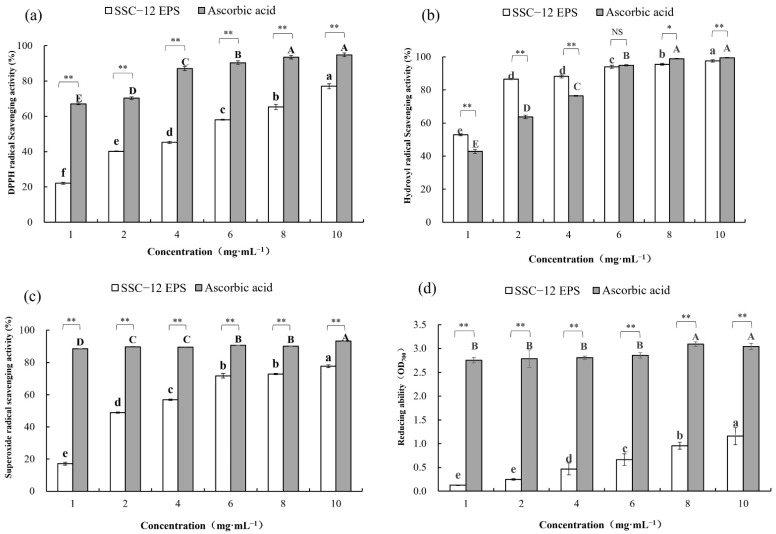
Antioxidant ability of SSC–12 EPS and standard antioxidant reagent ascorbic acid. SSC–12 EPS, EPS produced by SSC–12. Antioxidant ability includes DPPH radicalscavenging activity (**a**), Hydroxyl radicalscavenging activity (**b**), Superoxide radicalscavenging activity (**c**) and Reducing power (**d**). The values are expressed as mean ± SD of three independent analyses. Different lowercase letters indicate significant differences among SSC–12 EPS concentrations (*p* < 0.05). Different capital letters indicate significant differences among ascorbic acid concentrations (*p* < 0.05). The significance of SSC–12 EPS and ascorbic acid values at the same concentration was represented by “** *p* < 0.01, * *p* < 0.05, ^NS^ *p* > 0.05”.

As shown in Figure 4c, the hydroxyl radical-scavenging ability of SSC–12 EPS was higher than that of ascorbic acid, with 2 mg/mL of EPS (86.6%) exhibiting roughly 1.4-times higher scavenging activity than ascorbic acid (63.7%) at the same concentration. At low concentrations (1.0–4.0 mg/mL), the hydroxyl radicalscavenging ability of SSC–12 EPS was substantially stronger than that of ascorbic acid. At 4 mg/mL, the hydroxyl radical-scavenging ability of SSC–12 EPS (87.3%) was also higher than the EPS produced by *L. helveticus* MB2–1 (56.3%) [38]. However, the hydroxyl radicalscavenging ability of SSC–12 EPS (95.3%) was similar to that of EPS produced by *L. kimchi* SR8 (96.6%) at 8 mg/mL [33].

Our results indicated that EPS produced by SSC–12 has good antioxidant capacity and might serve as a good alternative to ascorbic acid. The degree of antioxidant capacity of EPS produced by different LAB differed, which may be attributed to the varying composition and structure, such as glycosidic linkages embodiment, functional group, and molecular weight [11]. For example, it has been reported that the EPS produced by *L. delbrueckii* ssp. *bulgaricus* SRFM–1 had more carboxyl functional groups than those produced by other LAB, which could provide an acidic environment to promote its hydrolysis and expose more hemiacetal hydroxyl groups for excellent antioxidant activity [39]. Moreover, a series of experiments proved that the EPS with lower molecular weight also showed higher antioxidant activity, which might be because low molecular weight can reduce the possibility of intramolecular hydrogen bonding and increase the exposure of active fragments to environmental reactions [11]. There are also some substituent groups that can affect the antioxidant activity of EPS. For example, sulfating modification can significantly improve the ability of the EPS to scavenge hydroxyl radicals and DPPH free radicals [24].

### 3.7. Antibacterial Ability

Pathogens can cause food to decay in vitro, while pathogenic bacteria in the gastrointestinal tract can cause gastrointestinal infections in vivo [11]. Antibiotics are widely used for the control of bacterial infections. However, as the phenomenon of drug resistance becomes a growing concern, the search for safe and effective antibacterial drugs has also become a focus for researchers. It has been reported that some EPSs from LAB have good antibacterial activity. For example, the EPS of *L. plantarum* HM47 isolated from human breast milk had a strong inhibitory effect on pathogenic *Escherichia coli* and *Salmonella typhimurium* in vitro [40]. The EPS of *L. fermentum* S1 isolated from traditional fermented Fuyuan pickle had good antibacterial activity against *E. coli* and *Staphylococcus aureus*, with the highest inhibition rates of 32% and 43%, respectively [41].

**Figure 5:**
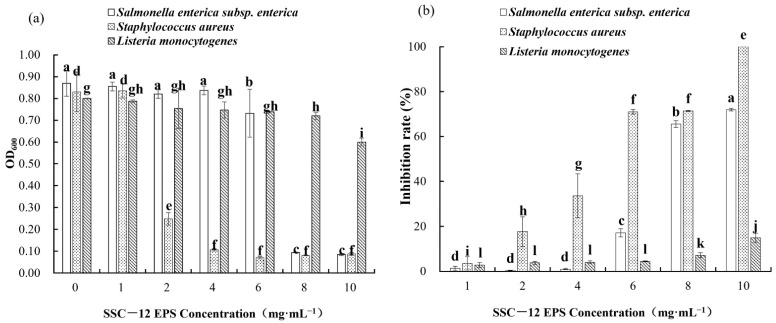
Antibacterial ability of SSC–12 EPS at different concentrations. SSC–12 EPS, EPS produced by SSC–12: The inhibitory ability on *Staphylococcus aureus*, *Salmonella enterica* subsp. *enterica,* and *Listeria monocytogenes* are determined by OD600 (**a**) and inhibition rate (**b**). The values are expressed as mean ± SD of three independent analyses. Different lowercase letters indicate significant differences among SSC–12 EPS concentrations of same indicator bacterial (*p <* 0.05).

The absorbance of a bacterial liquid indicates its turbidity degree. The higher the absorbance, the more the number of bacteria, and the determination of absorbance facilitated the qualitative analysis of the antibacterial property of SSC–12 EPS in this study. The inhibition rate of SSC–12 EPS was quantitatively analyzed by measuring the number of harmful bacteria cultivated in the culture medium after adding SSC–12 EPS. The inhibitory effect on *Staphylococcus aureus* and *Salmonella enterica* subsp. *enterica* increased with increase in SSC–12 EPS concentration, but the inhibitory effect on *Listeria monocytogenes* was not significant (Figure 5). Although the inhibitory effect of SSC–12 EPS on the three harmful bacteria was concentration-dependent, SSC–12 EPS had a considerable inhibitory effect on *Staphylococcus aureus*, *Salmonella enterica* subsp. *Enterica*, and *Listeria monocytogenes* at 2, 6, and 8 mg/mL, respectively. At 10 mg/mL, the inhibitory effect of SSC–12 EPS on *Staphylococcus aureus*, *Salmonella enterica* subsp. *Enterica*, and *Listeria monocytogenes* reached the maximum, which were 100%, 71.9%, and 14.9%, respectively. These results suggest that the SSC–12 EPS has the strongest inhibitory effect on *Staphylococcus aureus*, followed by *Salmonella enterica* subsp. *enterica*, and the worst inhibitory effect on *Listeria monocytogenes*. Liu et al. [1] observed that the EPS produced by *L. plantarum* WLPL04 had good inhibitory effect on *Staphylococcus aureus* and *Listeria monocytogenes* and its inhibitory ability increased gradually with increasing concentrations of EPS. At 2 mg/mL, the inhibitory rate of EPS produced by *L. fermentum* S1 against *Staphylococcus aureus* did not exceed 12% [41], while SSC–12 EPS reached 17.8% at the same concentration. When the EPS produced by *P. pentosaceus* M4 was 5 mg/mL [24], its inhibitory effect on *Staphylococcus aureus* reached 56.5%, which was similar to that of SSC–12 EPS.

Different types of EPS have varying inhibitory effects on harmful microorganisms. According to previous studies, the antibacterial mechanism of EPS might be attributable to the prevention of biofilm formation or destruction of membrane integrity and fluid soluble protein, which are mediated by signal molecules or sugar receptors [42,43]. The antibacterial ability of EPS was affected by its molecular framework, characteristic functional groups, and source of bacteria. For example, the EPS with carboxyl group often exhibited strong antibacterial activity because it could provide more lone electron pairs to strengthen the hydrogen bond interaction between molecules [11]. In addition, the EPS with a large number of decomposition escaping 1→4 linkages (para-substitution) often showed strong antimicrobial activity [8]. SSC–12 EPS had good antibacterial activity, providing a theoretical basis for its application in feed production and clinical treatment. However, the associated mechanism is unclear and needs further exploration.

## 4. Conclusions

In this study, a novel strain of EPS-producing strain SSC–12 isolated from silage was identified as *P. pentosaceus*. SSC–12 had strong vitality and fast EPS production rate. EPS produced by *P. pentosaceus* SSC–12 was a heteropolysaccharide and consisted of glucose (42.6%), mannose (28.9%), galactose (16.2%), arabinose (9.4%), and rhamnose (2.9%). The EPS had strong antioxidant ability and antibacterial ability. The present study identified a new strain of EPS-producing LAB, and the EPS showed good biological activity, which can potentially be applied in the feed, food, and pharmaceutical industries as well as in the development of new natural antibiotic substitutes.

## Figures and Tables

**Table 1:** The sources and EPS yield of 22 suspecting LAB strains screened from silage.

Source Silage	Strains	Yield (mg/L in MRS Broth, Mean ± SD)
Napier grass	SPP–1	59.3 ± 2.9 ^l^
	SPP–2	67.9 ± 4.9 ^kl^
	SPP–3	62.4 ± 5.1 ^l^
	SPP–5	87.5 ± 6.5 ^jk^
	SPP–6	202.5 ± 17.9 ^bc^
	SSP–9	172.6 ± 27.2 ^def^
Corn	SSC–1	51.0 ± 5.0 ^l^
	SSC–3	200.5 ± 16.3 ^bc^
	SSC–12	276.6 ± 12.1 ^a^
	SSC–16	110.6 ± 6.3 ^i^
	SSC–23	212.3 ± 10.5 ^b^
	SSC–48	143.7 ± 16.7 ^gh^
Orchardgrass	SDJ–3	30.5 ± 5.2 ^m^
	SDJ–6	188.5 ± 9.0 ^cd^
	SDJ–8	203.7 ± 8.2 ^bc^
	SDJ–16	131.1 ± 10.0 ^h^
Stylo	SS–9	90.5 ± 4.9 ^j^
	SS–17	181.7 ± 10.2 ^cde^
Soybean	SGM–9	202.9 ± 11.5 ^bc^
	SGM–16	164.8 ± 15.8 ^ef^
	SGM–18	176.8 ± 19.9 ^de^
	SGM–20	153.2 ± 7.3 ^fg^

Data with different letters within a column were significantly different at *p* < 0.05. The EPS yield in the table was mean ± SD of three tails.

**Table 2:** The monosaccharide composition of EPS produced by SSC–12.

Monosaccharide Name	Molar Ratio (%)
Glucose	42.7 ± 0.28
Mannose	28.9 ± 0.39
Galactose	16.3 ± 0.06
Arabinose	9.4 ± 0.36
Rhamnose	2.9 ± 0.02

The values are represented as mean ± SD (*n* = 3).

## Data Availability

All data generated or analyzed during this study are included in this article.
